# 

**Published:** 1849-07

**Authors:** 


No. II. JULY. Vol. V.

### BUFFALO MEDICAL JOURNAL AND MONTHLY REVIEW OF MEDICAL AND SURGICAL SCIENCE.

EDITED BY AUSTIN FLINT, M. D.

BUFFALO:
PUBLISHED BY JEWETT, THOMAS & CO.
Commercial Advertiser Buildings

1849.

